# Increased Serum Pigment Epithelium-Derived Factor in Women with Gestational Diabetes Is Associated with Type 2 Diabetes

**DOI:** 10.1155/2015/346938

**Published:** 2015-03-30

**Authors:** Tong-Huan Li, Chun-Jian Qiu, Xiao-Juan Yu, Dan-Dan Liu, Peng-Fei Zhou, Liang Wu

**Affiliations:** Department of Endocrinology, No. 81 Hospital of PLA, Nanjing 210002, China

## Abstract

*Background*. Pigment epithelium-derived factor (PEDF) is demonstrated to be elevated in diabetes patients. However, no reports have emerged in pregnant women with gestational diabetes mellitus (GDM). This study was undertaken to investigate serum PEDF levels in GDM women and to evaluate PEDF as a biomarker to predict diabetes postpartum. *Methods*. Serum PEDF concentration and clinical characteristics were detected in the pregnant women with GDM (*n* = 120) and without GDM (control group, *n* = 120). *Results*. PEDF levels were elevated in subjects with GDM versus controls. Univariate correlations showed that serum PEDF levels were positively correlated with fasting glucose and fasting insulin levels, respectively, and negatively correlated with adiponectin. Receiver operating characteristic (ROC) analysis demonstrated that the AUC of serum PEDF for diabetes mellitus in women postpartum was 0.893. *Conclusion*. Serum PEDF was elevated in pregnant women with GDM, which is probably an early detection marker for predicting development of GDM to diabetes mellitus.

## 1. Introduction

Gestational diabetes mellitus (GDM) is any degree of glucose intolerance with onset or first recognition during pregnancy, and the prevalence of GDM is rapidly increasing [1]. Most women with GDM revert to normal glucose metabolism after delivery of their babies; however, they are at risk of developing type 2 diabetes later in life as are their offspring. It has been reported that prevalence of the metabolic syndrome and type 2 diabetes in women with previous GDM is higher than in the general ones [2, 3]. The offspring of the women with GDM also have increased risk of metabolic syndrome or diabetes [4]. Therefore, GDM is an important public health concern, which threatens the health of pregnant women and their offspring.

Pigment epithelium-derived factor (PEDF) is first considered to be a natural extracellular component of the retina and its decreased level has been shown to participate in the pathogenesis of diabetic retinopathy [5, 6]. The production of circulating PEDF is mainly considered to be liver and adipose tissue [7]. In recent years, PEDF was reported to have potent anti-inflammatory, antiangiogenic, antioxidant, and microvascular protective properties [8–10]. Researchers also found that PEDF is associated with insulin sensitivity, diabetes mellitus, and its complications [11–13]. Clinical and epidemiological studies have proven that serum PEDF is increased in type 2 diabetes mellitus (T2DM) patients [14]. However, if it is good marker to predict the GDM development to diabetes after delivery is still unknown. In present study, the serum PEDF was detected at gestation and the predictive potential was evaluated in 240 pregnant women.

## 2. Methods and Materials

### 2.1. Subjects

The ethics committee of No. 81 Hospital of PLA approved this study, and all participants gave informed consent. 240 pregnant Chinese women were recruited at the time of antepartum screening for GDM in this study and a retrospective analysis was performed.

### 2.2. Definitions of GDM and Type 2 Diabetes

The diagnosis of GDM was performed as recommended by the American Diabetes Association [15]. Briefly, a 50 g OGCT was performed without prior fasting at 24–28 weeks of gestation. Women with a measured 1 h plasma glucose level above 7.8 mmol/L were considered to have a positive screening test and underwent a diagnostic 100-g OGTT. For the diagnostic 100-g OGTT, fasting plasma glucose levels were initially measured, followed by three plasma glucose measurements taken at hourly intervals (1, 2, and 3 h) after ingestion of a glucose load. Women with two or more plasma glucose levels as followings are defined to be GDM: fasting glucose of ⩾5.3 mmol/L, 1 h glucose of ⩾10.0 mmol/L, 2 h glucose of ⩾8.6 mmol/L, and 3 h glucose of ⩾7.8 mmol/L. At 3 months postpartum, OGTT was also performed in all subjects to diagnose T2DM. Criteria for the diagnosis of diabetes are as follows. (1) A1C ⩾ 6.5%: the test should be performed in a laboratory using a method that is NGSP certified and standardized to the DCCT assay. (2) FPG ⩾ 7.0 mmol/L is defined as no caloric intake for at least 8 h. (3) 2 h serum glucose ⩾ 11.1 mmol/L during an OGTT: the test should be performed as described by the World Health Organization, using a glucose load containing the equivalent of 75 g anhydrous glucose dissolved in water. (4) In a patient with classic symptoms of hyperglycemia or hyperglycemic crisis, a random plasma glucose ⩾11.1 mmol/L. In the absence of unequivocal hyperglycemia, criteria (1)–(3) should be confirmed by repeat testing.

### 2.3. Physical Body Assessment

Body weight and height were measured using an electronic weighing scale and stadiometer (HGM-200, Hangzhou, China). BMI was calculated as follows: body weight (kg)/[height (m) × height (m)].

### 2.4. Biochemical Analysis

All blood samples were obtained in the morning after the patients had fasted overnight at 24–32 weeks of gestation. Serum glucose, total cholesterol, triglycerides, HDL-c, LDL-c, and fasting insulin concentrations were determined with standard laboratory methods using an automatic biochemical analyzer (HF400; Shanghai, China). HOMA-IR was calculated as follows: (fasting serum glucose × fasting insulin)/22.5. serum adiponectin and PEDF concentrations were measured by ELISA kits (R&D Systems, USA) according to the operation instruction. The sensitivity in this assay was up to 18.75 pg/mL. The specificity for detection of PEDF was excellent, and no significant cross-reactivity or interference between PEDF and analogues was observed.

### 2.5. Statistical Analysis

Data were analyzed using the Statistical Package for the Social Sciences (SPSS, version 17.0; USA). The skewed variables were logarithmically transformed for statistical analysis. All data were expressed as means ± SD. Method of independent-samples* t*-test was used to compare means between GDM group and control group. A general linear model analysis was also performed and Pearson's correlation coefficients were used to examine relationships between PEDF and clinical characteristics. A receiver operating characteristic (ROC) curve was drawn to assess diagnostic accuracy; the number of GDM patients included to the ROC-AUC analysis was 110 (10 subjects were lost to followup). *P* < 0.05 was considered statistically significant.

## 3. Results

### 3.1. Subjects Characteristics

The subject characteristics are shown in Table 1. Age, week of gestation, prepregnancy BMI, total cholesterol, triglyceride, HDL-C, and LDL-C in GDM women were not significantly different compared with control (*P* > 0.05). The GDM women had higher serum levels of 0 h, 1 h, and 2 h glucose values during 75 g OGTT, fasting insulin, and HOMA-IR than that in control ones (*P* < 0.05). In addition, serum adiponectin level was markedly reduced and PEDF concentration was markedly elevated in GDM women versus control subjects.

### 3.2. Univariate Correlations

As shown in Table 2, serum PEDF level was positively correlated with fasting glucose, 1 h and 2 h glucose during 75 g OGTT (*P* < 0.05). Serum adiponectin value was negatively correlated with PEDF level (*P* < 0.05). However, PEDF concentration did not show an association with age, week of gestation, prepregnancy BMI, and blood lipids levels (*P* > 0.05). As shown in Table 3, glucose 1 h, glucose 2 h, and HOMA-IR remained in the multivariate linear regression analysis as independent predictors with *P* < 0.01.

### 3.3. ROC Curve Analysis

**Figure 1:**
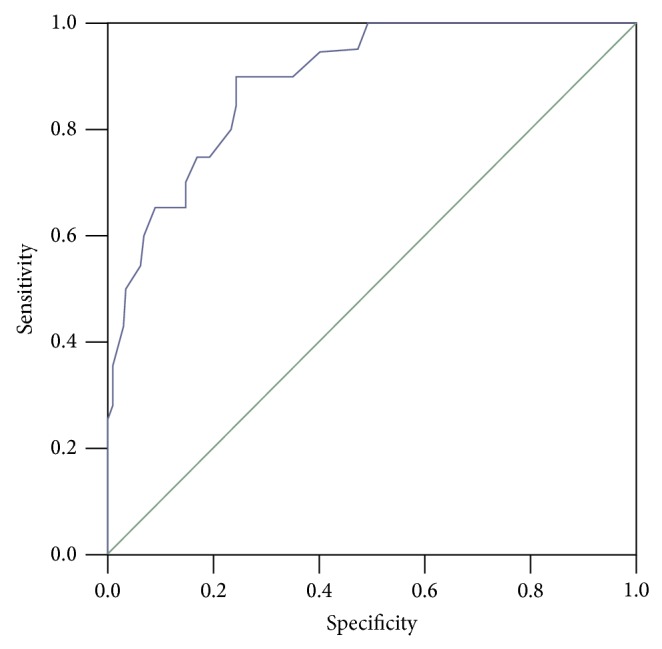
ROC curve showing the predictive probabilities of serum PEDF for type 2 diabetes mellitus. The number of GDM patients included to the ROC analysis was 110 (10 subjects were lost to followup).

3 months postpartum, OGTT was performed in all subjects and 20 GDM women were diagnosed to be T2DM. The diagnostic value of serum PEDF (detected in pregnant weomen) was evaluated by ROC analysis (Figure 1). The values of AUC, optimal cut-off value, sensitivity, and specificity for PEDF, respectively, were 0.893 (95% confidence interval: 0.853–0.933; *P* < 0.01), 4.23 *μ*g/mL, 0.900, and 0.667.

## 4. Discussion

Pigment epithelium-derived factor (PEDF) was first defined in conditioned medium of human retinal pigment epithelial cells as a factor with neuronal differentiating activity [5, 16]. In recent years, researchers found that PEDF is associated with diabetes mellitus and its complications [17].

Gestational diabetes mellitus (GDM) has an increasing prevalence during the last decade [18]. The precise mechanisms underlying GDM remain unclear, and gestational age, progestation BMI, and family history are included in the risk factors in development of GDM. GDM is once thought to be a simply impaired glucose tolerance temporarily associated with pregnancy [19]. However, it is a medical condition associated with long-term consequences for both maternal and fetal morbidity, including the delivery trauma, increase of diabetes mellitus, and obesity incidence [20, 21]. Therefore, prevention and reasonable control of GDM are important for the health of baby and mother.

There are emerging evidences to prove that PEDF is involved in microvascular complications, hyperglycemia, inflammation, and cardiovascular diseases [22]. Yamagishi et al. found that serum PEDF is elevated in proportion to the accumulation of the number of components of the metabolic syndrome [23]. PEDF has been demonstrated to inhibit the advanced glycation end product in adipocytes but causes insulin resistance in skeletal smooth muscle cells [7, 24]. In* in vivo* experiments, it is shown that PEDF could improve metabolic derangements by suppressing the inflammatory and oxidative reactions in adipose tissues of rats [25]. The scholars considered increased PEDF as a counter-system against obesity-related metabolic derangements. In present study, PEDF was measured at 24–32 weeks of gestation in all 240 pregnant women with or without GDM. The results showed that serum PEDF was elevated in GDM women compared to that in controls, and univariate correlations data in pregnant women demonstrated that serum PEDF level was positively related with fasting glucose and HOMA-IR, a marker of insulin resistance in high-risk patients for cardiovascular disease [26], which implied that GDM women had an abnormal serum PEDF. Increased PEDF was also observed in diabetes patients in previous studies [11, 14]. To our knowledge, this is the first study in pregnant women with GDM detecting serum PEDF level. Serum increased PEDF levels probably contributed to be a counter-system against metabolic abnormal in GDM women.

To further investigate the influences of serum PEDF during pregnancy on postpartum glucose levels, all subjects were followed up postpartum. At 3 months postpartum, 20 of 120 women previously with GDM were diagnosed to T2DM. The ROC curve analysis showed that the AUC of PEDF for T2DM of GDM women was 0.893, and the sensitivity and specificity for PEDF were 0.900 and 0.667, respectively, which implied that PEDF is probably an early marker for predicting T2DM after delivery in women previously with GDM. As described above, gestational age, progestation BMI, and family history of diabetes mellitus are important risk factors for GDM. In our study, the subjects in GDM group and control had no significant difference in gestational age and BMI, and all participants and their parents have no history of diabetes mellitus. We hold that it is necessary to take actions to prevent the development of T2DM in gestation if the serum PEDF concentration is abnormal. However, a larger sample size in different areas should be investigated to confirm this conclusion.

In summary, our study showed that serum PEDF was elevated in pregnant women with GDM, which is probably an early detection marker for predicting development of GDM to T2DM. Further studies are needed to explore the clinical prediction of PEDF detection in pregnant women.

## Figures and Tables

**Table 1:** Clinical and biochemical characteristics of gestational diabetes mellitus (GDM) patients and controls.

Parameter	GDM (*n* = 120)	Control (*n* = 120)	*P* value
Age (years)	30.14 ± 3.35	29.87 ± 3.07	0.421
Week of gestation	27.4 ± 1.5	26.8 ± 1.2	0.581
Weight (kg)	56.71 ± 5.48	55.98 ± 5.21	0.204
Height (cm)	158.42 ± 4.51	158.01 ± 4.27	0.618
Prepregnancy BMI (kg/m^2^)	23.49 ± 3.47	22.31 ± 2.71	0.178
Total cholesterol (mg/dl)	175.01 ± 21.31	183.14 ± 23.14	0.214
Triglyceride (mg/dl)	91.23 ± 51.31	84.12 ± 43.56	0.276
HDL-C (mg/dl)	47.19 ± 10.3	68.12 ± 9.6	0.105
LDL-C (mg/dl)	107.13 ± 20.18	97.18 ± 19.25	0.205
Glucose 0 h (mmol/L)	4.92 ± 0.91	4.21 ± 0.42	0.032
Glucose 1 h (mmol/L)	10.7 ± 1.72	7.12 ± 1.32	<0.01
Glucose 2 h (mmol/L)	9.02 ± 1.03	6.14 ± 1.26	<0.01
Fasting insulin (pmol/l)	68.13 ± 12.12	55.35 ± 7.12	0.037
HOMA-IR	2.67 ± 1.23	1.62 ± 0.67	<0.01
Adiponectin (mg/l)	12.34 ± 6.12	23.05 ± 7.12	<0.01
PEDF (*μ*g/ml)	5.02 ± 2.12	3.34 ± 1.23	<0.01

The difference of values between GDM patients and controls was performed by independent-samples *t*-test; *P* < 0.05 was considered to be significantly different; BMI: body mass index; HDL-C: high-density lipoprotein cholesterol; LDL-C: low-density lipoprotein cholesterol; HOMA-IR: homeostasis model assessment of insulin resistance; PEDF: pigment epithelium-derived factor.

**Table 2:** Univariate correlations with serum pigment epithelium-derived factor (PEDF) levels in gestational diabetes mellitus (GDM) patients.

Parameter	*r*	*P* value
Age	0.05	0.721
Week of gestation	0.02	0.621
Weight	0.08	0.435
Height	0.01	0.918
Prepregnancy BMI	0.17	0.08
Total cholesterol	−0.07	0.312
Triglyceride	−0.1	0.255
HDL-C	0.07	0.413
LDL-C	−0.04	0.578
Glucose 0 h	0.2	0.031
Glucose 1 h	0.37	<0.01
Glucose 2 h	0.34	<0.01
Fasting insulin	0.24	0.011
HOMA-IR	0.371	<0.01
Adiponectin	−0.412	<0.01

Univariate correlations were performed by a general linear model analysis and Pearson's correlation coefficients (*r*) were used to examine relationships between PEDF and clinical characteristics; *n* = 120; *P* < 0.05 was considered to be statistically related; BMI: body mass index; HDL-C: high-density lipoprotein cholesterol; LDL-C: low-density lipoprotein cholesterol; HOMA-IR: homeostasis model assessment of insulin resistance.

**Table 3:** Multivariate linear regression analysis of serum pigment epithelium-derived factor (PEDF) levels in gestational diabetes mellitus (GDM) patients.

Parameter	*Beta *	*P* value
Glucose 1 h	0.31	<0.01
Glucose 2 h	0.29	<0.01
HOMA-IR	0.332	<0.01

*n* = 120; *P* < 0.05 was considered statistically significant; HOMA-IR: homeostasis model assessment of insulin resistance.
